# Orthotopic Heart Transplantation in Patients with Univentricular Physiology

**DOI:** 10.2174/157340311797484259

**Published:** 2011-05

**Authors:** Guido Michielon, Adriano Carotti, Giacomo Pongiglione, Paola Cogo, Francesco Parisi

**Affiliations:** Dipartimento Medico-Chirurgico di Cardiologia Pediatrica Ospedale Pediatrico Bambino Gesù Roma, Italy

**Keywords:** Pediatric heart transplantation, fontan failure, univertricular physiology.

## Abstract

Parallel advancements in surgical technique, preoperative and postoperative care, as well as a better understanding of physiology in patients with duct-dependent pulmonary or systemic circulation and a functional single ventricle, have led to superb results in staged palliation of most complex congenital heart disease (CHD) [1]. The Fontan procedure and its technical modifications have resulted in markedly improved outcomes of patients with single ventricle anatomy [2,3,4]. The improved early survival has led to an exponential increase of the proportion of Fontan patients surviving long into adolescence and young adulthood [5]. Improved early and late survival has not yet abolished late mortality secondary to myocardial failure, therefore increasing the referrals for cardiac transplantation [6]. Interstage attrition [7] is moreover expected in staged palliation towards completion of a Fontan-type circulation, while Fontan failure represents a growing indication for heart transplantation [8]. Heart transplantation has therefore become the potential “fourth stage” [9] or a possible alternative to a high-risk Fontan operation [10] in a strategy of staged palliation for single ventricle physiology. Heart transplant barely accounts for 16% of pediatric solid organ transplants [11]. The thirteenth official pediatric heart transplantation report- 2010 [11] indicates that pediatric recipients received only 12.5% of the total reported heart transplants worldwide. Congenital heart disease is not only the most common recipient diagnosis, but also the most powerful predictor of 1-year mortality after OHT. Results of orthotopic heart transplantations (OHT) for failing single ventricle physiology are mixed. Some authors advocate excellent early and mid-term survival after OHT for failing Fontan [9], while others suggest that rescue-OHT after failing Fontan seems unwarranted [10]. Moreover, OHT outcome appears to be different according to the surgical staging towards the Fontan operation and surgical technique of Fontan completion [12].

The focus of this report is a complete review of the recent literature on OHT for failing single ventricles, outlining the clinical issues affecting Fontan failure, OHT listing and OHT outcome. These data are endorsed reporting our experience with OHT for failing single ventricle physiology in recent years.

## BACKGROUND

Neonatal palliation and staged reconstruction towards a Fontan-type circulation is currently feasible in most patients with complex CHD and single ventricle physiology. The concept of Fontan staging through a hemifontan or a bidirectional Genn shunting (BDG) has reduced the early mortality for Fontan completion, preventing the onset of diastolic ventricular dysfunction [13]. Recent publications report a 10 year survival after extracardiac Fontan completion varying between 85% and 93%. [2,3,4] . A recent large multicenter study on 321 patients who survived Fontan completion reported a 5-year transplant-free survival of 86% [14]. Technical evolution towards total cavopulmonary connection and the introduction of the fenestration concept have resulted in extended indication for the modified Fontan operation. Nevertheless interstage attrition prior to Fontan completion and relaxed Fontan criteria can result in early failure of a Fontan circuit [15]. Aside from the experience of Bailey [16], heart transplantation is usually considered as “the last resource” in the decision-making process, with a strong focus on the preservation of the native myocardium. Even in the ideal Fontan candidate, however, the instantaneous risk of death from cardiac failure progressively increases two years from surgery [17] and a steady decline in survival is documented between 5 and 7 years after Fontan completion [15].The attrition rate is known to be highest within the first six months after Fontan completion, but protein-losing enteropathy (PLE)[18], plastic bronchitis [19], arrhythmias, or progressive cardiac failure [15] can occur as potentially fatal complications of the Fontan procedures. Current evidence suggests that progressive alterations in the structure and function of the pulmonary vasculature may play a pivotal role in late Fontan failures [20]. Therefore single ventricle circulation appears to have a limited durability per se and myocardial dysfunction or failure/intolerance of Fontan physiology represent a growing indication for heart transplantation. Patients with single ventricle physiology are predicted to represent 70% to 80% of patients transplanted for congenital heart disease [21] in the near future The worldwide number of pediatric heart transplants has increased in recent years from an average of 384 OHT/year in the 1990s, to 428/year through the 2000s; in 2008, 491 pediatric heart transplants were reported to the Registry. Yet limited donor supply remains the major drawback, especially for small size recipients. The 13^th^ annual report report of the ISHLT [11] demonstrated a consistent and substantial improvement in Kaplan Meier survival over the last 2 decades, with a 65% 10-year survival for patients under 10 years of age. Nevertheless CHD still represents the leading independent predictor of 1-year mortality after pediatric heart transplantation, doubling the risk of death of OHT for non-congenital diagnosis. This occurs while 63% of OHT in infancy are accomplished on patients with CHD. Therefore, the issue is twofold. On one side CHD still represents an important independent predictor of mortality after OHT, and failing single ventricle is the most complex subgroup of CHD requiring OHT. On the other side there is a growing need for heart transplantation for failing single ventricles. Y*et al*l transplant candidates compete for the same donor pool, forcing the ethical issue of making the best use of a limited resource. The efficacy of OHT as a therapeutic option for failing single ventricles should ultimately consider two important points: modality of Fontan failure as an indication for OHT and optimal surgical staging of single ventricles towards Fontan completion versus heart transplantation. 

## MODALITIES OF FONTAN FAILURE

The Fontan circulation is based upon two fundamentals: adequate systolic/diastolic function of the single ventricle and unobstructed pulmonary pathway. The multifactorial mechanisms that contribute to Fontan failure undermine these two cornerstones for a successful Fontan palliation, nevertheless some are potentially correctable [22]. Primary palliative procedures strategy has a profound effect on both early and late success of Fontan circulation. Failure to provide early protection of the pulmonary vascular bed, delay in reducing volume loading of the single ventricle (systemic-to-pulmonary shunts, aorto-pulmonary collaterals), onset of atrioventricular or semilunar valve incompetence, do contribute to ventricular dysfunction, ultimately impacting Fontan outcome [23]. In addition, the Fontan circulation per se results in a chronically underloaded ventricle, following primary volume-loading palliative procedures [24,25]. The sequence of volume-loading followed by volume-unloading procedures promotes diastolic dysfunction, which can be worsened by any mechanism inducing ventricular hypertrophy, like new or recurrent obstructions in the systemic arterial pathway (i.e. sub-aortic stenosis, re-coarctation). Other failure mechanisms can result from anatomic substrates like heterotaxy [26,27] and especially asplenia syndrome, from anatomic lesions like stenosis in the pulmonary arterial pathway or pulmonary venous obstruction, or physiologic inefficiency of the Fontan pathway secondary to arrhythmias, like loss of sinus rhythm or atrial tachyarrhythmias. Most of these anatomic and physiologic drawbacks are potentially correctable [28] with Fontan revision [29], valve repair, pacing or catheter-based interventions, with the ultimate goal of preserving systolic and diastolic function of the single ventricle, while minimizing energy loss throughout the arterial and venous pulmonary pathway.

Conversely, other failure mechanisms are intrinsically related to the Fontan circulation itself, which sets the pulmonary capillary bed in series with the systemic, splanchnic and hepatic capillary bed, rendering the state of the pulmonary vascular bed critical to the Fontan circulation. Even mild increases in pulmonary vascular resistance are likely to have a negative impact on Fontan longevity. The underlying mechanism behind increased PVR in Fontan patients is not completely clear. Pulsatile pulmonary blood flow in normal hearts plays an important role in the reduction of PVR by passive capillary recruitment. When cardiac output increases, as in response to exercise, previously underperfused pulmonary vessels are recruited, thereby responding to increase in flow demand by decreasing PVR, resulting in a small increase in mean PAP relative to the increase in cardiac output. In the normal circulation most of the energy transmitted to the pulmonary arterial bed by right ventricular ejection is therefore committed to maintain patency of the distal pulmonary vascular bed [30]. In Fontan physiology, blood flow through the pulmonary arteries is non-pulsatile and largely driven by negative intrathoracic pressure and diastolic relaxation of the single ventricle. In the absence of pulsatile flow, recruitment of pulmonary vessels is reduced, potentially resulting in increased PVR. De Leval suggested that the intrinsic lower energy state of the pulmonary circulation in the Fontan circuit increases pulmonary vascular impedance and ultimately pulmonary afterload. The West zone two, or middle zone of the lungs, is probably smaller in the Fontan circulation [31], reducing pulmonary vascular compliance of the Fontan patient. Therefore the Fontan circulation per se promotes an increase in PVR, which can be worsen by increased pulmonary lymphatic pressure and chronic microembolic events. Pulsatile flow is also important in regulating the shear stress-mediated release of a number of endothelium-derived vasoactive molecules, and dysregulation of this mechanism may also lead to endothelium dysfunction. A number of endothelium-derived vasoactive factors have been implicated in the pathophysiology of the endothelial dysfunction characteristic of diseases such as pulmonary arterial hypertension [32] and their role has been investigated as a potential mechanism of increased PVR after Fontan completion. The combination of chronic impairment in the production of vasoactive mediators, such as endothelium-derived nitric oxide (NO) and prostacyclin, together with prolonged overexpression of vasoconstrictors such as endothelin 1, leads not only to abnormalities in vascular tone, but also to characteristic vascular proliferation and remodeling, endothelial dysfunction and associated increases in PVR. Studies of expression of these factors suggest that Fontan patients do present endothelial dysfunction. Patients with failing Fontan have been shown to over-express endothelial-NO synthase relative relative to patients with non-failing Fontan and normal controls [33]. Endothelin 1 is a potent vasoconstrictor with proliferative and hypertrophic effects on vascular smooth muscle cells. It appears that endothelin 1 levels are increased in Fontan patients in the acute postoperative phase and late after Fontan completion. Plasma endothelin levels are elevated in the acute postoperative course, and this increase correlate with elevation in PVR and central venous pressure early after Fontan completion, nevertheless these effects can be reversed by ultrafiltration. However, endothelin levels were found to be elevated even late after Fontan completion, especially in the proximal intra-acinar pulmonary arteries of patients with failing Fontan, compared with normal controls. Indeed, endothelin-1 single nucleotide G5665T polymorphism was found to be associated with a significant decrease in transplant-free survival for palliated single ventricles, with the majority of the decrement occurring in patients with HLHS [34].

Low cardiac output, excessive hypoxaemia, PLE and plastic bronchitis may all be clinical manifestations of increased PVR after Fontan completion. The development of pathologies such as protein-dispersion syndromes (protein-losing enteropathy, plastic bronchitis), aorto-pulmonary collaterals or pulmonary AV malformations cannot be frequently explained by hemodynamic pitfalls, and represent the most critical complications of the Fontan patient. The break in integrity of the enteric mucosal barrier seen in PLE affects a number of homeostatic systems [35]. Protein loss leads to reduced vascular oncotic pressure with interstitial and peripheral edema, ascites, pleural and pericardial effusions; loss of albumin leads to abnormalities in calcium metabolism; loss of coagulation factors further upsets an already abnormal cascade of coagulation; abnormal flow in lymphatic vessels, as a result of lymphatic engorgement, may lead to chylothorax, lymphopaenia and a relative immunodeficient state [36]. The pathophysiologic mechanism resulting in PLE is still enigmatic but two issues are believed to be involved: lower mesenteric oxygen delivery and inflammation. Gastrointestinal perfusion and oxygen delivery are reduced after Fontan completion [35]. This is demonstrated by an increased mesenteric vascular impedance measured by Doppler echocardiography in patients with Fontan circulation compared with age-matched normal controls. Patients with PLE after Fontan completion present significantly higher mesenteric vascular impedance and lower mesenteric-to-celiac flow ratio than those without PLE. Moreover flare of PLE symptoms in Fontan patients frequently occurs after acute viral infections, which implies an inflammatory mechanism [35,37]. Several studies demonstrated an increase in inflammatory markers in PLE, such as TNF-alpha, C reactive protein, cytokines which seem to play an important role in altering intestinal cell membrane permeability to intravascular proteins [20,35]. 

Plastic bronchitis is the other life-threatening complication of Fontan circulation [38]. Bronchial acellular casts forming within the airways can cause obstruction and determine asphyxiation. Clinical presentation is acute, and physical findings can dramatically and rapidly worsen. The break in integrity of the bronchial mucosa leads to leakage of proteinaceous material into the airways, and is probably secondary to lymphatic engorgement. Unlike PLE, hypoalbuminemia and edema may not occur, probably because relatively small amounts of protein are sufficient to cause significant airway obstruction.

Beyond Fontan fenestration [39,40] or aerosolized tissue plasminogen activator [41], lowering PVR even late after Fontan completion could prove to be beneficial per se. There are few observations describing the effects of PVR lowering drugs in the treatment of patients with failing Fontan. Recently Reinhardt [42] and Ozun [43] reported significant clinical improvement with the use of pulmonary vasodilators in PLE and plastic bronchitis after Fontan completion, therefore adding a useful therapeutical implement for failing Fontan circulation. Long-term treatment with bosentan improved symptoms, oxygen saturation, functional class, maximal and submaximal exercise capacity, Borg dyspnea index, mean PAP, pulmonary blood flow and PVR in a patient with plastic bronchitis, as reported by Apostolopuolou *et al* [44]. The effect of sildenafil on exercise-tolerance, ventricular function and health-related quality of life in children who have completed the Fontan procedure is currently under investigation. Lowering PVR in patients with failing Fontan circulation might be advantageous even as a bridge to heart transplantation [45], lowering mortality on the waiting list. We speculate that conditioning the pulmonary vasculature could be beneficial even before Fontan completion, and have successfully used pulmonary vasodilators in high-risk second stage procedures as well. 

If medical, surgical and catheter-based interventions are unable to improve symptoms, cardiac transplantation represents the only treatment option for the failed Fontan circulation [8, 9, 12]. 

## LISTING AND OHT OUTCOME

Bernstein *et al* [46] reported the outcome of listing for cardiac transplantation in 97 patients with failing Fontan referred at 17 Pediatric Heart Transplant Study centers in North America between 1993 and 2001. Mean age at listing was 9.7 years and the mean interval from Fontan completion to listing was 4.9 years. However 22% were less than 6 months after Fontan and 31% less than 1 year, which raises the question of poor indication to Fontan completion at first. Hazard function and competing outcomes analysis in this study suggests that most Fontan patients who die while waiting, will do so in the first 6 months after listing. Survival on the waiting list was 78% at 6 months with a 15.5% overall mortality rate. Patients who were younger of 4 years of age at listing, status 1, patients who had shorter interval since Fontan or were ventilator-dependent, were more likely to die on the waiting list. Specifically, 33% of patients who were less than 6 months from their Fontan died on the waiting list, accounting for a dismal 40% survival at 6 months on the waiting list for early Fontan failure. Over 50% Fontan patients listed for OHT were status 1. At 6 months on the waiting list, the probability of receiving a transplant was similar for status 1 (65%) and status 2 (68%), however the probability of death before a transplant was 22% in status 1 and 5% in status 2. 

Actuarial OHT survival for Fontan diagnosis was 77% at 1 year and 67% at 5 years, significantly lower compared with 91% 1-year and 81% 5-year OHT survival for non-congenital diagnosis. Overall mortality for OHT after Fontan failure was 33% . This data suggests that the increased risk in Fontan patients is predominantly in the early period after transplantation. There was indeed a trend in the Fontan group toward a higher rate of early graft failure (17%), infections (30%) and acute hemorrhage/operative complications (9%) as cause of death, compared with other CHD and non-CHD diagnosis. Early graft failure can be attributable to the presence of preformed antibodies (PRA) secondary to use of homograft patches and multiple blood transfusions that increase the risk of pre-sensitization, but could also reflect a fixed element of PVR that is unmasked with the introduction of normal pulmonary blood flow after OHT. It is erroneous to assume that because a Fontan patient is alive, the pulmonary vascular resistance is low enough to tolerate a OHT. Intolerance to Fontan physiology with prolonged pleural effusions, ascites, lymphopenia and protein dispersion can predispose to life-threatening infections that are triggered by post-OHT immunosuppression. However this study documented that 73% patients with PLE survived on the waiting list for OHT, 76% of those receiving a heart survived after OHT with PLE resolution in all OHT survivors. 

Lamour *et al* [47] conducted a study on behalf of the Cardiac Transplant Registry Database in association with the Pediatric Heart Transplant Study on 488 patients with CHD (107 Fontan and 381 non-Fontan) undergoing OHT between 1993 and 2002. Lamour demonstrated that Fontan patients had a 71% 1-year and 60% 5-year OHT-survival, significantly lower than 83% 1-year and 74% 5-year OHT-survival documented in non-Fontan patients with CHD. Specifically having a previous Fontan procedure increased 8.6 times the relative risk of death after OHT in this cohort. Conditional survival in patients who survived the first 3 months after transplant was not different between Fontan patients and other non-Fontan CHDs, indicating that the increased risk of death is likely related to peri-transplant issues. This study suggested that the complexities of OHT after Fontan completion are not only associated with the technical challenges related to the individual anatomy, multiple earlier palliative procedures and pulmonary artery distorsion, but also to difficulty in evaluating PVR and an increased propensity for infection and bleeding in Fontan patients. These studies infer that OHT in failing Fontan carries substantial early risk, higher than in other CHD [48] and even higher than non-CHD. OHT cannot be considered as a rescue therapy for patients who fail the Fontan procedure, neither a viable option for younger patients who are early Fontan failures. In contrast, OHT remains a very good option for older children and adolescents who are late Fontan failures. 

These data are in agreement with our previous report on OHT outcome for end-stage CHD. In a cohort of 43 patients undergoing OHT for CHD between 1988 and 2002. Michielon *et al* [10] verified that single ventricle physiology entailed substantial early mortality compared with other CHD, accounting for a 67% early OHT-survival in single ventricles as opposed to 94% in other CHD. Michielon first identified bidirectional Glenn shunting as the best bridge to heart transplantation in failing single ventricle physiology [12], therefore suggesting OHT as a valid alternative to high-risk Fontan completion. Indeed OHT transition from a BDG stage enabled 100% early and late OHT survival, as opposed to 33% early and 5-year survival for OHT after Fontan failure. This data was noteworthy since it represented a single center experience with systematic and uniform approach to single ventricle physiology, therefore avoiding the typical bias of multi-institutional studies, such as surgical strategy of Fontan staging, technique of Fontan completion and referral criteria for transplantation. Indeed in our experience Fontan completion was systematically prepared by intermediate staging with BDG, regardless ventricular morphology, and all Fontan completions were accomplished by extracardiac technique with conduit interposition between the inferior vena cava and the central pulmonary arteries, therefore allowing the best energy preservation pattern of Fontan circulation, as confirmed by physical and mathematical models, and the lowest risk of atrial arrhythmias [2]. In other words, Fontan failure in this cohort was secondary to predominant failure of Fontan physiology, not to untreatable arrhythmias, atrial thrombosis or primary ventricular dysfunction. We concluded that OHT should be considered in the decision-making process as an alternative to Fontan completion in high-risk candidates, since rescue OHT after failing Fontan seemed unwarranted. 

Jayakumar [21] reported the combined New York, Philadelphia and Columbus experience on 35 patients with failing single ventricles undergoing OHT between 1984 and 2001. Surgical technique of Fontan completion was not elucidated, but 57% of patients underwent OHT for ventricular dysfunction, and only 43% for failed Fontan physiology. The 1-year OHT-survival after Fontan completion was 63% as opposed to 90% 1-year OHT-survival after Glenn staging. Jayakumar stated that this difference in survival was not statistically significant, but reported that 9 out of 10 early deaths occurred in the Fontan failure subgroup as opposed to only one death for OHT after Glenn shunting. Jayakumar confirmed previous reports on high attrition in the early post-OHT period for failing Fontans, with over 70% of deaths occurring within a week of transplant mainly due to operative hemorrhage and infections, therefore underlying the challenging technical and immunosuppressive issues involved in this subgroup of patients. This data confirmed the experience of Michielon *et al*. with bidirectional Glenn shunting as the best bridge to OHT in failing single ventricle physiology [12].

Gamba *et al* [9] reported better OHT outcome after failing Fontan in a cohort of 14 patients undergoing OHT between 1990 and 2002. Their 1-year OHT survival was 86% and the 5-year survival was 77%. However the mean age at OHT in this cohort was 17.2 years, and the mean interval between Fontan completion and OHT was almost 10 years. Therefore this experience mainly refers to OHT for late Fontan failure, which is known to have better overall outcome. Indeed most Fontan completion in this cohort were accomplished by atriopulmonary connections, and single ventricle dysfunction, arrhythmias and atrial thrombosis represented the most common indication for OHT (57%). Nevertheless this report confirmed the benefit of OHT on PLE recovery, although a 5-year mean follow-up does not allow for conclusive statements.

These reports suggest that the modes of presentation for the failed Fontan Fontan circulation is twofold: failed Fontan physiology and ventricular dysfunction. Failed Fontan physiology, such as refractory ascites, pleural effusions, PLE, plastic bronchitis may occur in the setting of relatively preserved ventricular function. Characterization of ventricular function in patients with indeterminate or right dominant ventricular morphology is largely subjective and measurement of the end-diastolic pressure in underloaded ventricles with low-output and poor transit of systemic venous blood through the lungs may be misleading. Based on these findings, Griffiths and Del Nido [49] identified the indication to OHT performed at CHB between 1994 and 2008 in failed Fontan with impaired ventricular function versus patients with preserved ventricular function but failed Fontan physiology. PLE or plastic bronchitis were present in over 80% patients with preserved ventricular function and in no patients with impaired ventricular function group. The impaired ventricular function group had significantly lower cardiac index, SvO2 and significantly higher SVR compared to the preserved ventricular function group. One year actuarial survival after OHT was 88.9% in impaired ventricular function vs 56.2% in preserved ventricular function. One year survival in preserved ventricular function was similar to one year survival in non transplanted patients, raising the question of the efficacy of OHT in this subgroup of patients. 

Improvements in OHT survival according to year of transplant have been reported by most centers as well as by the Registry of the International Society for Heart and Lung Transplantation [10, 11, 21, 48]. There continues to be a small trend towards an increasing number of pediatric heart transplants reported to the Registry, from an annual average of 384 transplants in the 1990’s, to 428 in the 2000’s to 491 in 2008. Earlier year of transplant was found to be a significant risk factor for mortality. This finding is mirrored in the overall experience of OHT for CHD [10, 48] and OHT for failed single ventricle physiology as well. Jayakumar [21] reported a 64% 5-year OHT-survival for failing single ventricles transplanted before 1995, and a 77% 5-year OHT-survival for transplants performed between 1996 and 2001. We report an 8-year survival of 79.6% in 25 patients undergoing OHT for failing single ventricles between 2003 and 2010. The 5-year survival for OHT in failing Fontan was 66%. These results compare favorably with our previous 33% 1-year survival for failing Fontans undergoing OHT before 2002. Ventricular dysfunction was the dominant mechanism of late extracardiac Fontan failure (80% patients), while early Fontan failure was mainly due to failing Fontan physiology in the setting of relatively preserved ventricular function (80% patients). Fontan status remained a risk factor of mortality after OHT while bidirectional Glenn staging was confirmed as the best bridge to heart transplant, with 100% 5-year survival after OHT, even in our most recent experience. Early Fontan failure represented the only independent predictor of mortality after OHT, with and odds ratio of 13.1 and a dismal 20% 1-year survival, while excellent outcome (90% 5-year survival) can be expected in OHT candidates for late Fontan failure without significant end-stage comorbidities (hepatic cirrhosis, chronic malnutrition). 

BDG is therefore advocated as the best bridge to OHT, while early Fontan failure is mostly associated with failure of Fontan physiology, which renders rescue OHT outcome unwarranted. We could speculate that Fontan take-down in early Fontan failure could not only improve overall survival but also OHT outcome. Take-down Fontan should theoretically decompress the splanchnic venous congestion assuring optimal ventricular filling and cardiac output, at the expense of systemic oxygen desaturation. Down-staging to bidirectional Glenn should reproduce the excellent results of OHT after BDG. However this issue is not straightforward. The overall experience with Fontan takedown has been discouraging, with an early mortality rate approaching 50%. Knott-Craig reported [50] a 62.5% mortality rate after early Fontan take-down on over 700 modified Fontan operations performed at the Mayo Clinic over a 16-year interval. A more recent experience of the Boston’s Children group reported a 45% mortality rate in 53 Fontan take-downs performed between 1979 and 2006 [51]. Take-down performed within a month from Fontan completion (acute take-down) was associated with highest mortality rate (51%). Indeed Almond [51] retrospectively verified the presence of correctable abnormalities in 44% of early Fontan failures. Therefore out of the 29 take-down survivors, only 3 patients underwent OHT within 14 years from Fontan take-down, with no early mortality. We believe that early Fontan take-down remains the only life-saving surgical option for early Fontan failure, but in our limited experience we could not demonstrate a definitive advantage of take-down for early Fontan failure on immediate post-OHT outcome, and verified a 50% mortality in rescue OHT after Fontan take-down. 

In conclusion, OHT is an effective therapy for failing single ventricles. OHT outcome has improved in recent years, with an expected 5-year survival approaching 80% in the overall experience with failing single ventricles. Fontan status remains a risk factor of mortality after OHT with an expected 5-year survival barely approaching 70%, partially related to the evidence of pulmonary vascular disease after OHT for Fontan circulation failure [52]. The attrition is highest in OHT after early Fontan failure, with a 50% 1-year survival in the best reported series. Mortality frequently occurs in the early post-OHT period, for predominant infective complications. Tailoring of immunosuppressive therapy is a crucial issue in these patients since they are immunocompromized for their Fontan failure. Pre-sensitization for previous blood transfusions and liberal use of homograft material is very common [53], puzzling the balance between immunosuppression and risk of life threatening infections. Excellent OHT outcome and 90% one-year survival is predicted for late Fontan failures, with predominant ventricular dysfunction and without significant comorbidities such as hepatic cirrhosis or chronic malnutrition. PLE is a catastrophic complication of Fontan completion, but can be improved by OHT [9,21,54,55], although mortality on the waiting list can be as high as 40%. Moreover long-term fate of PLE after OHT has not been completely elucidated, and recurrence of PLE even after OHT has been reported. Early diagnosis of other life-threatening Fontan complications, such as plastic bronchitis, is crucial to allow for effective medical therapy, by a combination of potent pulmonary vasodilator and fibrinolitic agents [41-45] and early referral for OHT. Bidirectional Glenn staging currently represents the best bridge to heart transplant in failing single ventricles, but Fontan take-down and down-staging to BDG does not warrant comparable OHT outcome as BDG at first. This caveat underlines the critical point of optimal referral criteria for both Fontan completion and OHT in single ventricle physiology. OHT should be considered as an alternative to Fontan completion in the decision-making algorithm for high-risk Fontan candidates, since rescue OHT after early Fontan failure is unwarranted. In the near future, incorporation of a mechanical assist device such as an axial flow pump in the Fontan circulation may prove to be beneficial, either as a bridge to recovery, possibly converting the high risk patient into a relatively good candidate for the Fontan circulation, or as a bridge to transplant, therefore reducing attrition on the transplant list and bridging the worse patients to successful heart transplantation. 
